# Dynamic Mechanical Properties and Modified Material Constitutive Model for Hot Forged Ti_2_AlNb over Wide Ranges of Temperature and Strain Rate

**DOI:** 10.3390/ma17112572

**Published:** 2024-05-27

**Authors:** Liangliang Li, Xin Pan, Yongliang Zhang, Jianwei Mu, Jinfu Zhao, Xiangmin Dong, Zhifeng Liu

**Affiliations:** 1 Key Laboratory of CNC Equipment Reliability, Ministry of Education, Jilin University, Changchun 130025, China; liangleejob@163.com; 2 Key Laboratory of Advanced Manufacturing and Intelligent Technology for High-End CNC Equipment, Changchun 130025, China; 3 Innovation Research Institute, Shenyang Aircraft Corporation, Shenyang 110850, China; panx001@avic.com (X.P.); zhangyl009@avic.com (Y.Z.); mu.jw@avic.com (J.M.); 4 Key Laboratory of High Efficiency and Clean Mechanical Manufacture of MOE, School of Mechanical Engineering, Shandong University, Jinan 250061, China; sduzhaojinfu@sdu.edu.cn; 5 Department of Mechanical Engineering, Hebei Petroleum University of Technology, Chengde 067000, China

**Keywords:** Ti_2_AlNb, quasi-static, detached Hopkinson compression bar, intrinsic modeling

## Abstract

In this paper, the stress–strain curves of Ti_2_AlNb are established based on uniaxial impact tests over wide ranges of temperature and strain rate. The Ti_2_AlNb exhibited the work hardening effect but did not show an obvious yield stage during a quasi-static compression test. In the SHPB test, an obvious temperature softening effect was found, the strain rate strengthening effect was detected when the strain rate was 4000–8000 s^−1^, and the strain rate softening effect was detected in the range of 8000–12,000 s^−1^. A function describing the effect of strain rate on the strain rate strengthening parameters under various temperatures was proposed to modify the basic J-C constitutive model. The relative errors between the experimental measured value and predicted values in various experimental conditions with a modified J-C model were less than 5.0%. The results verified that the modified J-C model could accurately describe the dynamic mechanical properties of Ti_2_AlNb at high temperatures and strain rates. The research could help to illustrate the cutting mechanism and finite element simulation of Ti_2_AlNb alloy.

## 1. Introduction

Titanium–aluminum alloys based on intermetallic compounds (e.g., α-Ti3Al, γ-TiAl, δ-TiAl_3_, etc.) possess high high-temperature strength, strong oxidation resistance, good creep resistance, and excellent organizational stability due to the combined effect of the metallic and covalent bonds [1,2,3]. However, the development of titanium–aluminum alloys is limited by poor room-temperature brittleness. Therefore, Ti_2_AlNb alloys based on ordered rhombohedral phases (O) have been developed in the past decades, which were first discovered by Banerjee in his 1988 toughening experiments of Ti_3_Al-based alloys [4]. Compared to TiAl-based alloys, Ti_2_AlNb alloys possess better room-temperature plasticity, fracture toughness, as well as crack extension resistance, and possess better high-temperature strength and oxidation resistance [5,6]. In addition, the density of Ti_2_AlNb-based alloys is reduced by about 40% compared to iron-based and nickel-based high-temperature alloys without affecting the material high-temperature properties [7]. Ti_2_AlNb is potential aviation material with broad application prospects to replace nickel-based high-temperature alloys due to its superior properties.

D The morphology and corresponding mechanical properties of Ti_2_AlNb alloys are highly dependent on the fabrication process (e.g., casting, forging, powder metallurgy, etc.) and heat treatment procedures (e.g., deformation, solution treatment, recrystallization, stress relief annealing, aging, etc.). Ti_2_AlNb-based alloys fabricated by conventional casting methods have large grain sizes and poor properties. The large difference between the melting points of the Al and Nb elements could induce compositional segregation and uneven microstructure during the smelting process of Ti_2_AlNb alloy. Thermomechanical processing (TMP) is necessary to refine the grain size and eliminate defects of smelted Ti_2_AlNb alloy, such as in hot forging and hot rolling [8]. Ti_2_AlNb is a typical difficult-to-machine material due to its excellent room- and high-temperature mechanical properties. High cutting forces and high local cutting temperatures could be induced to cause severe tool wear during the cutting process of Ti_2_AlNb alloy. Therefore, it is worth researching the cutting process of Ti_2_AlNb alloy. Finite element simulation is a low-cost and high-efficiency methodology for illustrating the cutting mechanism of Ti_2_AlNb alloy. The prediction accuracy of finite element simulation is dependent on the established Johnson–Cook (J-C) model of Ti_2_AlNb alloy [9,10].

Many studies on the methods of establishing the J-C model have been conducted for nickel-based alloys and titanium alloys [11,12,13,14]. These current methods could guide the establishment of the J-C model of Ti_2_AlNb alloys. Hou et al. [15] obtained stress–strain curves with the quasi-static compressive and split Hopkinson pressure bar (SHPB) experiment results of Ti-6Al-4V. The material constitutive model was modified with the function proposed to describe the coupling between temperature and strain. Tian et al. [16] conducted similar experimental processes of GH2132 nickel-based alloy. The strain rate sensitivity coefficient was modified with a bivariate quadratic function of temperature and strain rate for the established J-C model of GH2132. Ling et al. [14] established the J-C model considering recrystallization softening for nickel-based powder metallurgy superalloys. Lin et al. [17] conducted experimental research on the mechanical behavior of 5A06 aluminum alloy in three different processing and heat treatment states at 25–500 °C and strain rates of 10^−4^–10^−3^ s^−1^. Based on the J-C constitutive model, the constitutive model parameters of the materials in each state were fitted through experimental data, and the strain rate strengthening term in the Johnson–Cook constitutive model was modified. Zhou et al. [18] conducted AZ91D compression experiments at a strain rate of 400–1000 s^−1^, and the results showed that the strain rate sensitivity of AZ91D magnesium alloy increased with increasing strain rate. Hu et al. [19] tested the dynamic mechanical behavior of V-5Cr-5Ti vanadium alloy at a strain rate of approximately 3000 s^−1^ at temperatures ranging from 15–1100 °C, and the results showed that the J-C model can accurately describe the dynamic mechanical behavior of vanadium alloy. It was found that necessary modifications should be conducted for the J-C models according to the characteristic stress–strain curves of the experimental material.

In the past decade, the constitutive models for Ti2AlNb-based alloys with various TMP also have been researched by some researchers. He et al. [20] established the Arrhenius and J-C constitutive models for the high-temperature deformation of hot-rolled Ti-22Al-25Nb (at.%) based on uniaxial tensile tests. They found that the Arrhenius model was suitable for relatively low strain rate deformation. The J-C model was more suitable for wide ranges of strain rates. The strain softening effect on the flow stress should be considered within the experimental temperature ranges of 930–990 °C. Xue et al. [21] carried out a room-temperature compression test of Ti_2_AlNb alloy with ultrasonic amplitude range of 0–31 µm and strain rate of 0.001–0.125 s^−1^. The Johnson-Cook (J-C) constitutive model was established with similar methods as shown in the literature. Sim et al. [22] conducted the isothermal uniaxial compression test of the fine-grained Ti_2_AlNb-based alloy fabricated by mechanical alloying and subsequent spark plasma sintering in the deformation temperature range of 950–1070 °C and the strain rate range of 0.001–1 s^−1^. He et al. [23,24] illustrated that the dynamic flow stress of Ti_2_AlNb is sensitive to the competition of the thermal softening effect with strain rate hardening effect. Wang et al. [25] conducted the uniaxial tension experiments of hot-rolled Ti-22Al-23Nb-2(Mo, Zr) alloys at both room (RT, 28 °C) and elevated temperatures (500, 550, and 650 °C). They found that the peak stress was significantly relevant to the test temperature. The stress softening effect should be considered in the modified J-C model. In the above research, the maximum strain rate is only 10^−3^, which is an order of magnitude different from the range of cutting strain rates. At the same time, there is still a lack of corresponding research on whether there is a coupling effect in parameter components and the quantitative relationship between coupling effects.

Knowledge of the dynamic mechanical properties of Ti_2_AlNb based on the accurate Johnson–Cook (J-C) model could guide the accurate finite element simulation for metal cutting. In this paper, the dynamic mechanical properties of hot forged Ti_2_AlNb are analyzed with stress–strain curves based on quasi-static compressive and uniaxial impact tests over wide ranges of temperature (25–800 °C) and strain rate (4000–12,000 s^−1^). The J-C constitutive model was established and modified with consideration of the thermal and stress softening effect, etc. The relative errors between the experimental measured value and predicted values in various experimental conditions were obtained. The results verified that the modified J-C model could accurately describe the dynamic mechanical properties of Ti_2_AlNb at high temperatures and strain rates.

## 2. Material and Methods

### 2.1. Materials

**Figure 1:**
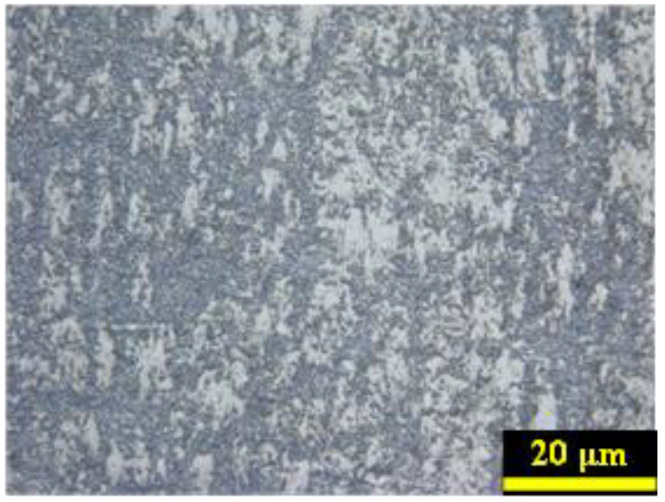
Metallographic microstructure of hot forged Ti_2_AlNb.

Ti2AlNb alloy was selected for experiments, and the mechanical properties of Ti2AlNb under different temperatures and strain rates were measured using quasi-static compression devices and split Hopkinson pressure bar (SHPB) devices (Zongde company, Jinan, CHN). The original Ti_2_AlNb alloy was melted three times in an electric arc furnace (Institute of metal sciences, Beijing, China). The specimens were obtained after the hot forging process. The preheating treatment was carried out according to specific technical documents. The solid solution treatment of the forged Ti_2_AlNb was maintained for 1–4 h with the temperature range of 900–1100 °C. The aging treatment of the forged Ti_2_AlNb was maintained for 12–24 h with the temperature range of 750–870 °C. The chemical element composition of hot forged Ti_2_AlNb alloy is listed in Table 1. The tensile strength of hot forged Ti_2_AlNb alloy at various temperatures was ≥1000 MPa (room temperature), ≥800 MPa (650 °C), ≥680 MPa (750 °C), and ≥580 MPa (800 °C). Figure 1 shows the metallographic microstructure of hot forged Ti_2_AlNb. The microstructure of Ti_2_AlNb alloy was detected as the bimodal or tristate organization of *α*_2_ + O + B_2_. The particles of α_2_/O were uniformly distributed for the hot forged Ti_2_AlNb alloy.

### 2.2. Quasi-Static Compression Test

The cylindrical specimen of hot forged Ti_2_AlNb alloy was machined with a diameter of 8 mm and the length *L*_0_ = 10 mm [26]. The geometric accuracy and surface quality of specimens met the requirements, which was guaranteed by the precision turning and grinding.

**Figure 2:**
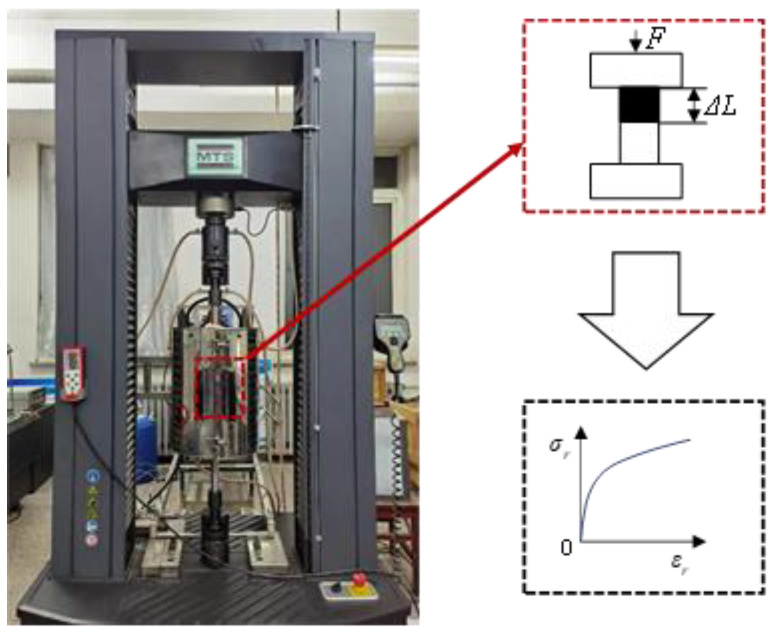
Quasi-static compression test setup.

As shown in Figure 2, the quasi-static compression tests were carried out with an electronic universal testing machine of the type MTS (BY82 Hainan Lanpu Electromechanical Technology Co., Danzhou, China). The strain rate of the unidirectional compression test at room temperature was set at 0.001 s^−1^, which was determined with Equation (1) [17]. The compressive rate was calculated as 0.6 mm/min according to the specimen height. The varied compressive force *F* and displacement of the specimen were captured with a pressure transducer and displacement transducer, respectively. Then, the true stress–strain curves could be determined according to the sample size as depicted in Equations (2) and (3) [15]. Three repeated compression tests were carried out for the machined specimen to reduce the error.

(1) $$\overset{•}{\varepsilon }=\frac{d\varepsilon }{dt}=\frac{1}{dt}\left(\frac{dh}{h}\right)=\frac{1}{h}\frac{dh}{dt}=\frac{v}{h}$$

(2) $$\sigma_{r}=\frac{F}{A_{0}}\left(1-\frac{\Delta L}{L_{0}}\right)$$

(3) $$\varepsilon_{r}=-\ln\left(1-\frac{\Delta L}{L_{0}}\right)$$

where $\overset{•}{\varepsilon }$ is the strain rate. ε is the strain. *v* is the compression rate. *h* is the specimen height. $\sigma_{r}$ is the true stress. *A*_0_ is the initial cross-sectional area. $\varepsilon_{r}$ is the true strain.

### 2.3. Split Hopkinson Pressure Bar Tests

The cylindrical specimen of hot forged Ti_2_AlNb alloy was machined with a diameter of 2 mm and length of 2 mm in order to obtain high strain rates in SHPB tests [27]. The specimens were handled by EDM wire cutting, surface grinding, centerless grinding, and polishing.

**Figure 3:**
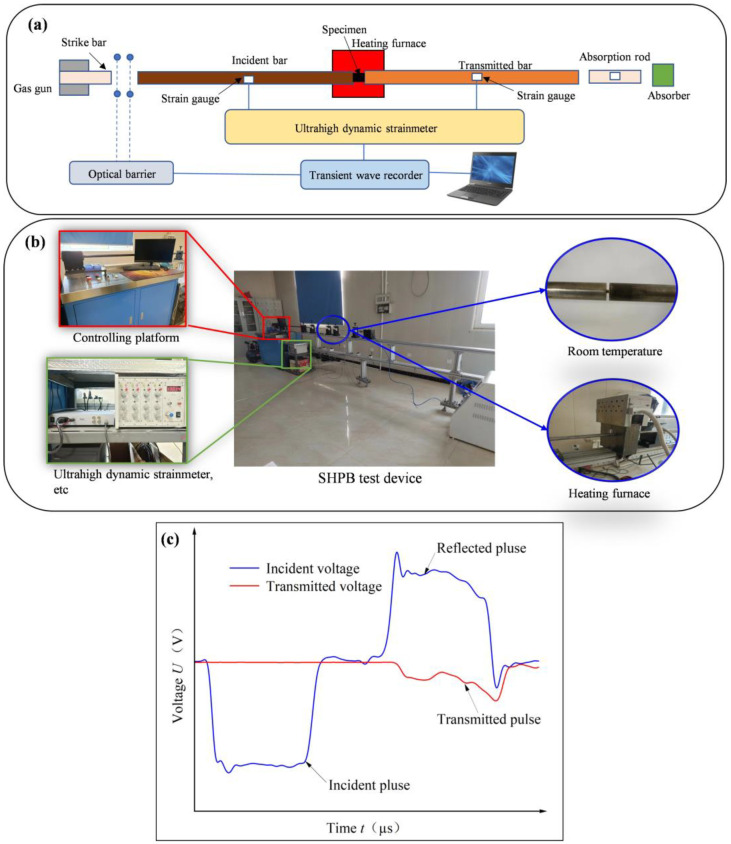
SHPB test: (**a**) schematic diagram; (**b**) device; (**c**) typical voltage waves.

Figure 3a depicts the schematic structure of the SHPB test setup. Figure 3b shows the SHPB test setup. Figure 3c shows the typical voltage waves. The diameters of the incident and transmitted bars were 10 mm. The material of the incident and transmission bars was 18 Ni. The maximum heating temperature of the heating furnace was 1000 °C. With consideration of the strain rate and cutting temperature in the low- and medium-speed cutting processes, the strain rates of the SHPB test were assumed as 4000 s^−1^, 6,000 s^−1^, 8000 s^−1^, 10,000 s^−1^, and 12,000 s^−1^. The experimental temperatures of SHPB tests were taken as 25 °C, 200 °C, 400 °C, 600 °C, and 800 °C, respectively. The strain rates of the SHPB test were adjusted by the impact velocity of an impact rod based on the specific pressure of an air pump. Two sets of strain gauges were applied to record the raw data of the incident, transmitted, and reflected pulses. The high-temperature-resistant lubricant MoS_2_ was utilized to decrease the friction at the contact surfaces between the specimen and incident and transmission rods. Each set of tests was repeated three times to reduce the error. Typical pulse data obtained during the tests are shown in the illustration of Figure 3a.

## 3. Results and Discussion

### 3.1. True Stress–Strain Curves of Hot Forged Ti_2_AlNb Alloy in Quasi-Static Compression Tests

**Figure 4:**
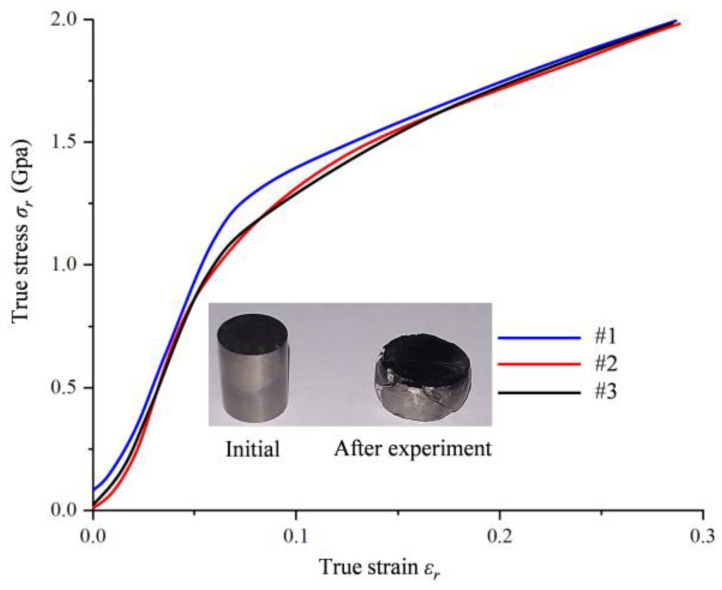
True stress–strain curves of hot forged Ti_2_AlNb alloy in quasi-static compression tests.

Figure 4 depicts the true stress–strain $\sigma_{r}-\varepsilon_{r}$ curves of hot forged Ti_2_AlNb alloy. The three sets of test data overlap well. The true stress increased basically linearly with the increase in strain in the initial compressive loading stage. The true stress increased slowly when the true strain exceeded 0.04. The material deformation of Ti_2_AlNb showed the apparent elasticity stage and reinforcement stage, but no obvious yielding stage was found. In the plastic deformation (strengthening) stage, the true stress increased monotonically with the increase in true strain. It illustrated that a significant work hardening phenomenon of hot forged Ti_2_AlNb alloy existed in the compression tests.

### 3.2. Deformation and Microstructure of Hot Forged Ti_2_AlNb in SHPB Tests

**Figure 5:**
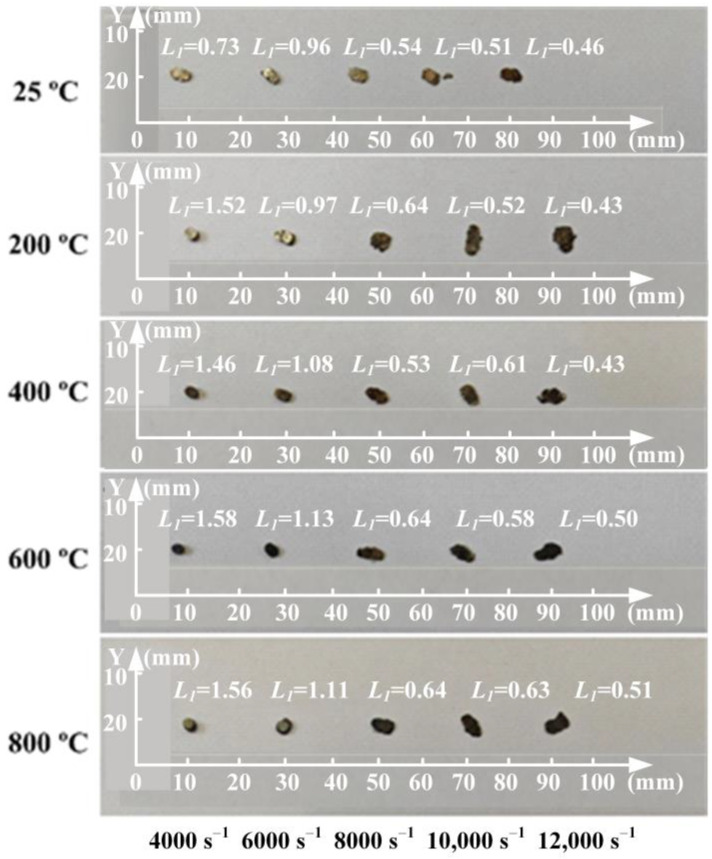
Deformed specimens after SHPB tests.

The deformed specimens after SHPB tests are summarized in Figure 5. The height values of the deformed specimens are also listed in Figure 5. The specimens were only pressed into a round cake shape without fracture when the applied strain rate was less than 6000 s^−1^ within a temperature range of 25–800 °C. The specimen was fractured and deformed into a flat shape when the strain rate was more than 8000 s^−1^. When the test was conducted at a temperature of 800 °C and stress rate of 12,000 s^−1^, the height of deformed specimen *L_1_* became 25.5% of the initial height of specimen *L_0_*, and obvious compression deformation was detected for the specimen.

**Figure 6:**
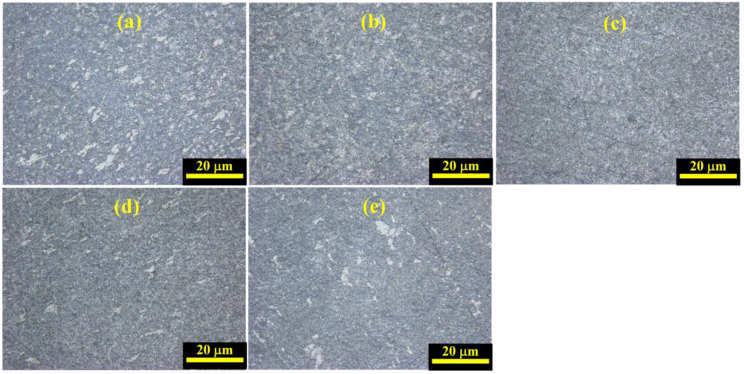
Variation of metallographic microstructures of Ti_2_AlNb alloys after SHPB tests with varied temperature values at strain rate 4000 s^−1^: (**a**) 25 °C, (**b**) 200 °C, (**c**) 400 °C, (**d**) 600 °C, (**e**) 800 °C.

**Figure 7:**
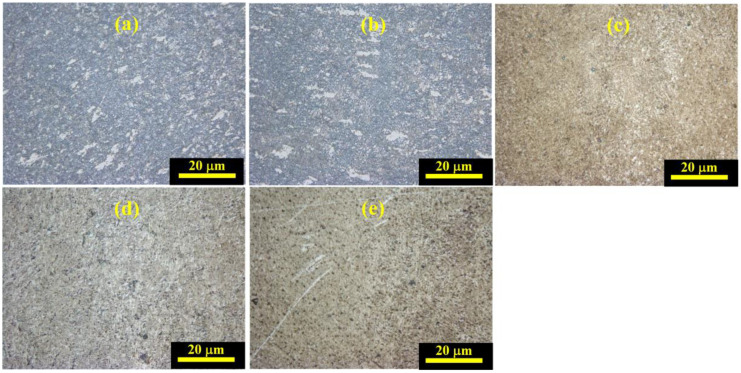
Variation of metallographic microstructures of Ti_2_AlNb alloys after SHPB tests with varied strain rates at temperature value of 25 °C: (**a**) 4000 s^−1^, (**b**) 6000 s^−1^, (**c**) 8000 s^−1^, (**d**) 10,000 s^−1^, (**e**) 12,000 s^−1^.

Figure 6 depicts the variation of metallographic microstructures of Ti_2_AlNb alloys after SHPB tests with varied temperature values at a strain rate of 4000 s^−1^. Compared to the initial metallographic microstructure of Ti_2_AlNb alloys before SHPB tests, grain refinement and recrystallization were found due to the dynamic impact. In addition, the equiaxial organization was obviously reduced and transformed into lamellar organization. The variation of metallographic microstructures of Ti_2_AlNb alloys after SHPB tests with varied strain rates at a temperature of 25 °C is summarized in Figure 7. The metallographic microstructures of Ti_2_AlNb alloy were transformed from bimodal organization into lamellar organization when the strain rate was more than 8000 s^−1^. In addition, the grain refinement and recrystallization phenomenon was also found.

### 3.3. Stress–Strain Curves of hot Forged Ti_2_AlNb Alloy in SHPB Tests

**Figure 8:**
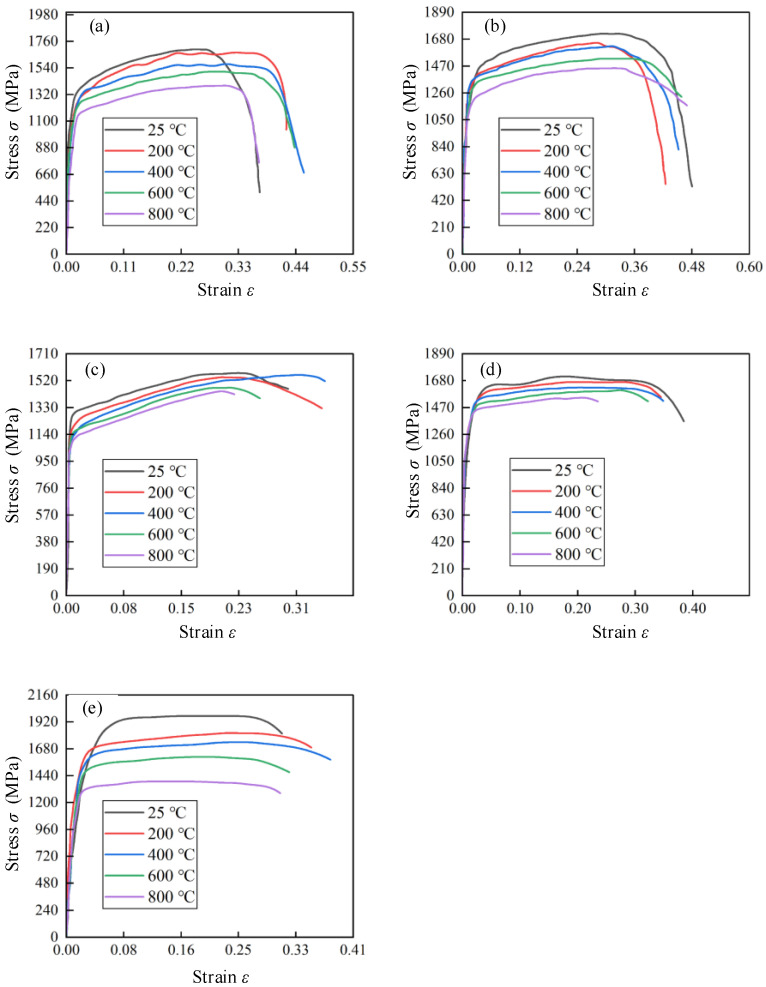
Stress–strain curves of Ti_2_AlNb alloys at specific strain rate: (**a**) 4000 s^−1^, (**b**) 6000 s^−1^, (**c**) 8000 s^−1^, (**d**) 10,000 s^−1^, (**e**) 12,000 s^−1^.

**Figure 9:**
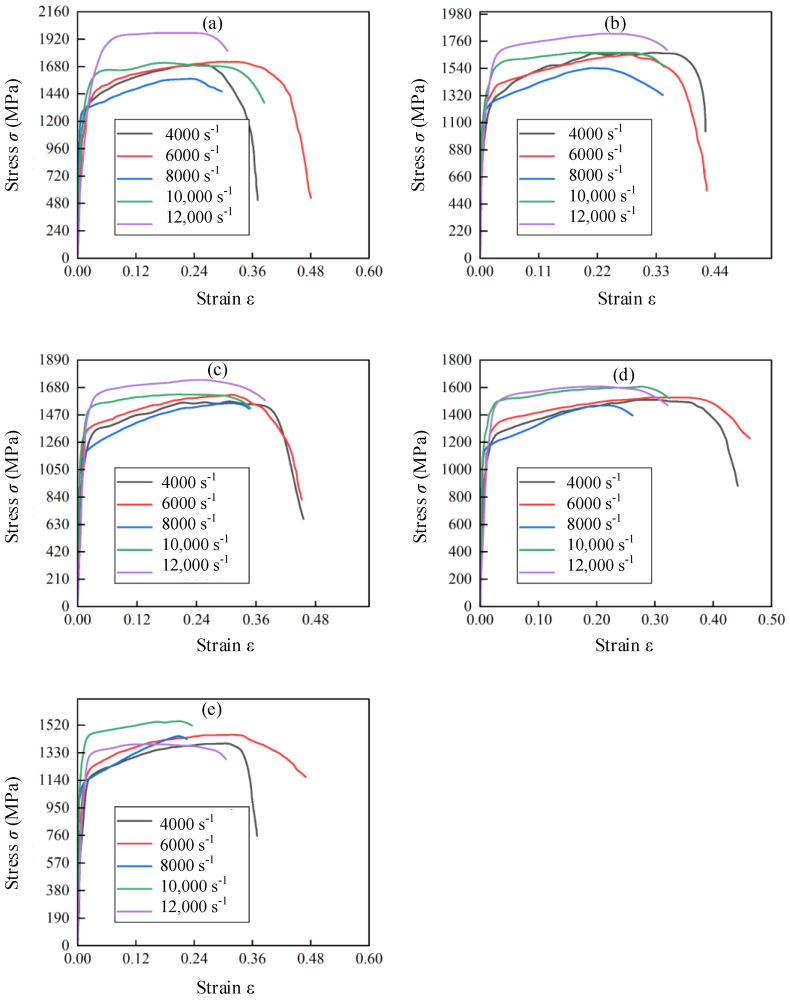
Stress–strain curves of Ti_2_AlNb alloys at specific temperature: (**a**) 25 °C, (**b**) 200 °C, (**c**) 400 °C, (**d**) 600 °C, (**e**) 800 °C.

Figure 8 depicts the stress–strain curves of Ti_2_AlNb alloys within a strain rate range of 4000–12,000 s^−1^. The significant temperature softening effect of Ti_2_AlNb alloy was detected. The stress was decreased with the increase in temperature in the plastic flow stage at the same strain rate. Figure 9 hows that the stress in the plastic flow stage showed a uniform decrease with the increase in the test temperature when the strain rate was 4000 s^−1^, 6000 s^−1^, 8000 s^−1^, or 10,000 s^−1^. The gradient of the stress decrease increased suddenly between different temperatures when the strain rate increased to 12,000 s^−1^, which indicated that the temperature softening effect of Ti_2_AlNb was affected by the strain rate. The material internal organization showed a critical state of change within a certain temperature interval, such as phase transformation and dynamic recrystallization. The large stress decrease occurred when the critical temperature of this state was reached.

Figure 9 shows the stress–strain curves of Ti_2_AlNb alloys within a temperature range of 25–800 °C. It was observed that the stress of Ti_2_AlNb alloy was not evidently changed with the increase in strain rate at a specific temperature. Weak strain rate sensitivity could be determined. The strain rate strengthening effect was detected within the strain rate range of 4000–8000 s^−1^. The stress gradually increased with the increase in strain rate. It was related to the dislocation density of Ti_2_AlNb alloy gradually increasing with the increase in strain rate. The dislocation entanglement, cross-cutting, and other obstacles appeared to increase the resistance of dislocation movement and the deformation resistance. Therefore, the strain rate strengthening effect was induced. However, the strengthening effect did not last for a long time when the strain rate continued to increase. The heat generated in the rapid deformation process could be concentrated in the deformation position. The heat could not be dissipated in time, which induced a serious adiabatic temperature rise. The evaluated temperature rise meant that the thermal softening effect would exceed the strain rate strengthening effect. Therefore, the overall strain softening effect was detected within the strain rate range of 10,000–12,000 s^−1^.

## 4. Establishment and Verification of Modified J-C Constitutive Model

### 4.1. Establishment of Basic J-C Constitutive Model

The basic J-C constitutive model was determined as shown in Equation (4) [28]. The three factorials from left to right of the J-C constitutive model represented the strain hardening effect, strain rate strengthening effect, and temperature softening effect, respectively. According to the conditions of this quasi-static compression test, $\overset{•}{\varepsilon_{0}}$, $\theta_{m}$, and $\theta_{r}$ were taken as 0.001 s^−1^, 1700 °C, and 25 °C, respectively.

(4) $$\sigma =\left(A+B\varepsilon_{p}^{n}\right)\left(1+C\ln\frac{\overset{•}{\varepsilon }}{\overset{•}{\varepsilon_{0}}}\right)\left[1-{\left(\frac{\theta -\theta_{r}}{\theta_{m}-\theta_{r}}\right)}^{m}\right]$$

where σ is the material plastic stress. $\varepsilon_{p}$ is the equivalent plastic strain. $\overset{•}{\varepsilon }$ and $\overset{•}{\varepsilon_{0}}$ are the equivalent plastic strain rate and reference strain rate, respectively. θ, $\theta_{m}$, and $\theta_{r}$ are the current temperature, the melting temperature, and the reference temperature. *A*, *B*, *n*, *C*, and *m* are the yield limit, strain hardening rate, strain hardening index, strain rate sensitivity coefficient, and the temperature softening index at the reference strain rate and the reference temperature, respectively.

The yield limit *A*, strain hardening rate *B*, and strain hardening index *n* could be determined with Equation (5) based on the Ludwik model with consideration of the strain hardening effect [29]. The stress at which 0.2% of the plastic strain occurred was taken as the yield limit *A*. *A* could be determined as 1019.4 MPa according to the true stress–strain $\sigma_{r}-\varepsilon_{r}$ curves of hot forged Ti_2_AlNb alloy in the quasi-static compressive test as depicted in Figure 4. Equation (5) could be taken logarithmically to obtain Equation (6) [30]. Equation (6) could be seen as a straight line with slope *n* and intercept ln*B*. The true stress–strain $\sigma_{r}-\varepsilon_{r}$ curves of hot forged Ti_2_AlNb alloy in the quasi-static compressive test were utilized to finish the linear fitting process when the strain was more than 0.04. *n* and *B* were determined as 0.8384 and 2777.75 MPa, respectively.

(5) $$\sigma =A+B\varepsilon_{p}^{n}$$

(6) $$\ln\left(\sigma -A\right)=n\ln\varepsilon_{p}+\ln B$$

**Figure 10:**
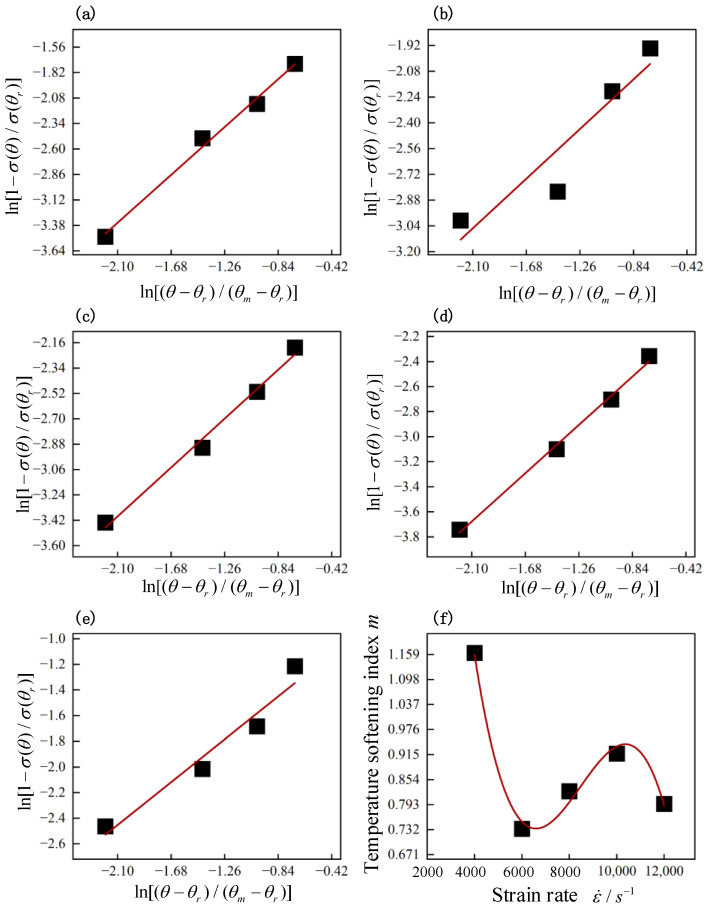
Fitted plots of m at specific strain rates. (**a**) 4000 s^−1^, (**b**) 6,000 s^−1^, (**c**) 8000 s^−1^, (**d**) 10,000 s^−1^, (**e**) 12,000 s^−1^. (**f**) *m* values vs. strain rate.

The strain rate sensitivity coefficient *C* and the temperature softening index *m* could be obtained based on the stress–strain $\sigma_{r}-\varepsilon_{r}$ curves of hot forged Ti_2_AlNb alloy in SHPB tests. The ratio of the stress $\sigma \left(\theta \right)$ at the test temperature *θ* to the stress $\sigma \left(\theta_{r}\right)$ at the reference temperature $\theta_{r}$ was given by Equation (7) when the strain rates were assumed to be the same. Equation (7) could be shifted and transformed into Equation (8). Equation (8) could be regarded as a proportional function with slope *m*, and its independent variable is $\ln\frac{\theta -\theta_{r}}{\theta_{m}-\theta_{r}}$ and dependent variable is $\ln\left[1-\frac{\sigma \left(\theta \right)}{\sigma \left(\theta_{r}\right)}\right]$. The plastic strains of hot forged Ti_2_AlNb alloy at varied strain rates are shown in Figure 8. The medium value of plastic strain was taken to represent the intermediate strain in the plastic deformation stage. Table 2 lists the medium value of plastic strain of hot forged Ti_2_AlNb alloy at varied strain rates. The strain value was determined at the medium value of plastic strain. The temperature softening index *m* was fitted according to the various strain rate and temperature values as shown in Figure 10. The fitted temperature softening index *m* at various strain rates is listed in Table 3.

(7) $$\frac{\sigma \left(\theta \right)}{\sigma \left(\theta_{r}\right)}=1-{\left(\frac{\theta -\theta_{r}}{\theta_{m}-\theta_{r}}\right)}^{m}$$

(8) $$\ln\left[1-\frac{\sigma \left(\theta \right)}{\sigma \left(\theta_{r}\right)}\right]=m\ln\frac{\theta -\theta_{r}}{\theta_{m}-\theta_{r}}$$

*m* at the strain rate of 4000 s^−1^ was relatively large, at about 1.59, 1.40, 1.26, 1.45 times the strain rate values of 6000 s^−1^, 8000 s^−1^, 10,000 s^−1^, and 12,000 s^−1^. It was indicated that the temperature softening coefficient *m* was correlated with the strain rate, which was in agreement with the results obtained in Section 3.2 and Section 3.3. The temperature softening coefficient *m* was corrected to be a univariate quadratic function with the strain rate as in Equation (9). The fitted m-$\overset{•}{\varepsilon }$ curves are depicted in Figure 10f. *m*_0_ = 4.811, *m*_1_ = −0.00157, *m*_2_ = 1.95 × 10^−7^, and *m*_3_ = −7.67 × 10^−12^ fitted well.

(9) $$m=m_{0}+m_{1}\overset{•}{\varepsilon }+m_{2}{\overset{•}{\varepsilon }}^{2}+m_{3}{\overset{•}{\varepsilon }}^{3}$$

**Figure 11:**
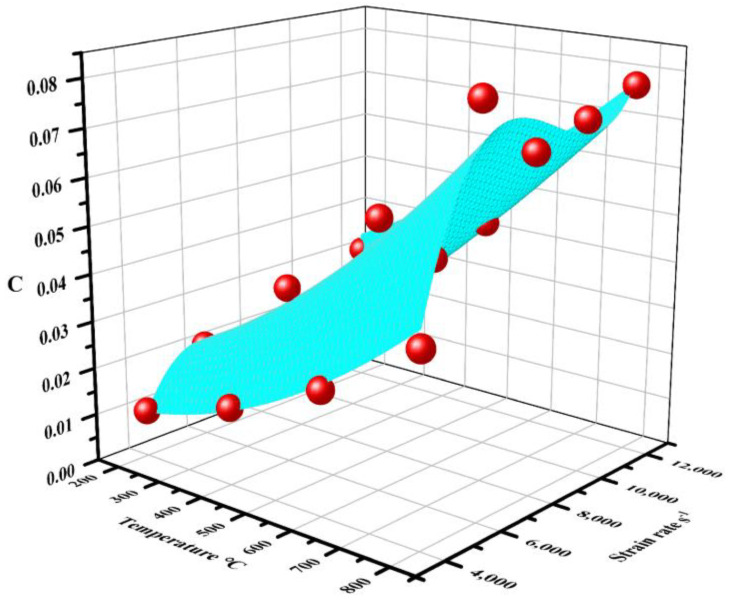
The fitting relationship between parameter C and temperature, strain rate.

Equation (4) could be transformed into Equation (10) to determine the parameter *C*. The strain rate sensitivity coefficient *C* at specific temperatures and strain rates could be calculated with the specific temperature, strain rate, and the fixed equivalent plastic strain $\varepsilon_{p}$ = 0.1 as summarized in Table 4. The parameter *C* could be modified as a function of temperature and strain rate as shown in Equation (11).

(10) $$C=\left\{\left.\frac{\sigma }{\left(A+B\varepsilon_{p}^{n}\right)\left[1-{\left(\frac{\theta -\theta_{r}}{\theta_{m}-\theta_{r}}\right)}^{m}\right]}-1\right\}\right.\frac{1}{\ln\frac{\overset{•}{\varepsilon }}{\overset{•}{\varepsilon_{0}}}}$$

(11) $$C=C_{0}+C_{1}\theta +C_{2}\overset{•}{\varepsilon }+C_{3}\theta^{2}+C_{4}\theta \overset{•}{\varepsilon }+C_{5}\overset{•}{\varepsilon^{2}}+C_{6}\theta^{2}\overset{•}{\varepsilon }+C_{7}\theta \overset{•}{\varepsilon^{2}}+C_{8}\overset{•}{\varepsilon^{3}}$$

where the fitting coefficients are obtained as C_0_ = −0.12872, C_1_ = −1.3561 × 10^−4^, C_2_ = 6.61193 × 10^−5^, C_3_ = 7.59744 × 10^−8^, C_4_ = 4.10412 × 10^−8^, C_5_ = −9.72203 × 10^−9^, C_6_ = −3.2756 × 10^−12^, C_7_ = −2.2184 × 10^−12^, C_8_ = 4.3993 × 10^−13^. The fitting surface is shown in Figure 11, and fitting error *R*_2_ was 0.9404.

### 4.2. Modification of Basic J-C Constitutive Model

The strain hardening rate *B* was only based on the strain rate in the quasi-static compression tests. The effects of strain rates in SHPB tests on parameter *B* also should be considered. In fact, the strain hardening rate *B* was a parameter which was varied with the strain rate. With consideration of the of strain rate on parameter *B*, the strain hardening rate in the plastic deformation stage was calculated as shown in Table 5 at specific strain rates in quasi-static compression and SHPB tests at room temperature.

Table 5 showed that the strain hardening rate was sensitive to the strain rate. The strain hardening rate in the quasi-static compressive test was significantly higher than the strain hardening rate in SHPB tests. The strain hardening rate decreased slowly with the increase in strain rate in SHPB tests. In order to characterize the relationship between the strain hardening rate *B* and strain rate, the relevant parameters were assumed to be dimensionless. The relationship between the strain hardening rate *B* and strain rate was fitted as in Equation (12).

(12) $$y=0.5+0.496e^{-\frac{1}{2.11\;\times \;10^{7}}\frac{\dot{\varepsilon }}{0.001}}$$

Equation (12) was transformed into Equation (4), and the modification of the basic J-C constitutive model was finished. The modified J-C constitutive model within the temperature range of 25–800 °C and the strain rate range of 0.001–12,000 s^−1^ is shown in Equation (13). The other parameters referred to in Equation (13) are listed as Equations (14)–(16), respectively.

(13) $$\sigma =\left(1013.4+B\varepsilon_{p}^{0.64}\right)\left(1+C\ln1000\overset{•}{\varepsilon }\right)\left[1-{\left(\frac{\theta -25}{1675}\right)}^{m}\right]$$

(14) $$B=2777.75\times (0.5+0.496e^{-\frac{1}{2.11\;\times \;10^{7}}\frac{\dot{\varepsilon }}{0.001}})$$

(15) $$m=4.811-0.00157\overset{•}{\varepsilon }+1.95\times 10^{-7}{\overset{•}{\varepsilon }}^{2}-7.67\times 10^{-12}{\overset{•}{\varepsilon }}^{3}$$

(16) $$\begin{array}{c} C=-0.1287-1.3561\times 10^{-4}\theta +6.61193\times 10^{-5}\overset{•}{\varepsilon }+7.59744\times 10^{-8}\theta^{2}+4.10412\times 10^{-8}\theta \overset{•}{\varepsilon }\\ -9.72203\times 10^{-9}\overset{•}{\varepsilon^{2}}-3.2756\times 10^{-12}\theta^{2}\overset{•}{\varepsilon }-2.2184\times 10^{-12}\theta \overset{•}{\varepsilon^{2}}+4.3993\times 10^{-13}\overset{•}{\varepsilon^{3}} \end{array}$$

### 4.3. Verification of Modified J-C Constitutive Model

**Figure 12:**
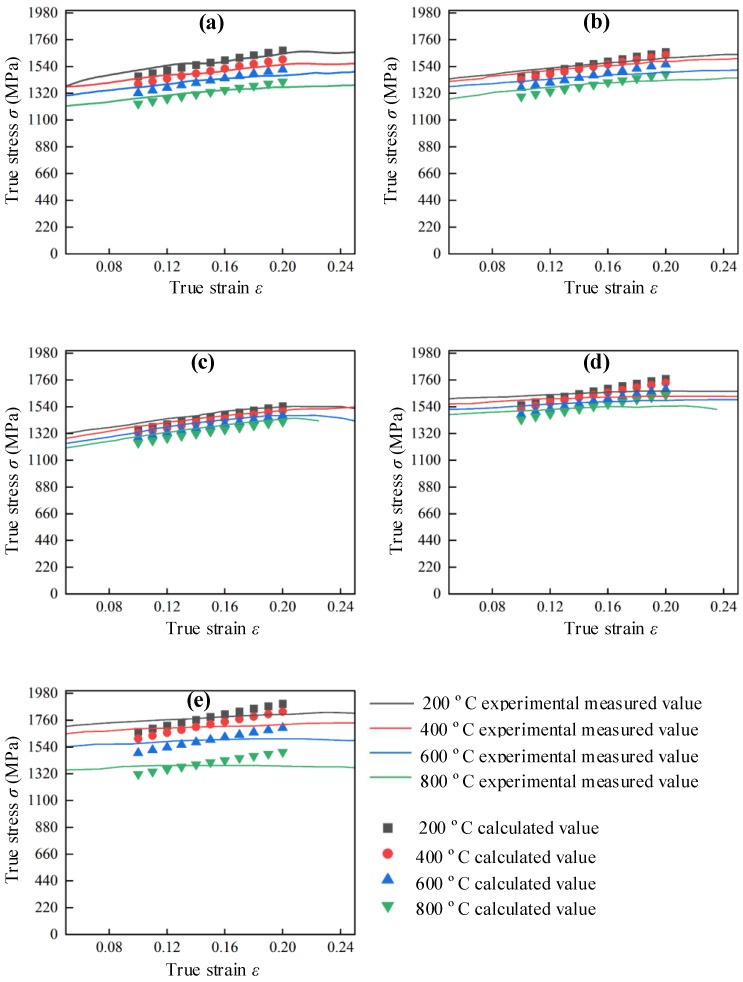
Experimental measured and calculated values of Ti_2_AlNb stress–strain curves at specific strain rates. (**a**) 4000 s^−1^, (**b**) 6000 s^−1^, (**c**) 8000 s^−1^, (**d**) 10,000 s^−1^, (**e**) 12,000 s^−1^.

The stress–strain relationship for hot forged Ti_2_AlNb alloy could be obtained according to Equation (13). In order to verify the accuracy of the modified J-C constitutive model, the calculated values were compared with the experimental measured values within the range of strain of 0.1–0.2 as shown in Figure 12. It could be found that the error between the calculated values and the experimental measured values increased with the increase in strain rate. The calculated values had good agreement with the experimental measured values. The relative error between the experimental measured stress values and the calculated values could be determined with Equation (17).

(17) $$\Delta =\frac{1}{n}\sum_{i=1}^{n}\left|\left.\frac{x_{i}-y_{i}}{x_{i}}\right|\right.\times 100\%$$

where *n* is the number of sampling points. *x_i_*, *y_i_* are the experimental measured stress value and calculated stress value at the *i*th sampling point, respectively.

Table 6 summarizes the relative errors between the experimental measured stress values and the calculated values at a specific strain rate and temperature. The maximum value of relative error was obtained as 5.0% at a temperature of 600 °C and a strain rate of 12,000 s^−1^. The relative errors in the other conditions were all less than 4.7%. It was verified that the modified J-C constitutive model could describe the dynamic mechanical properties of hot forged Ti_2_AlNb alloys well.

**Figure 13:**
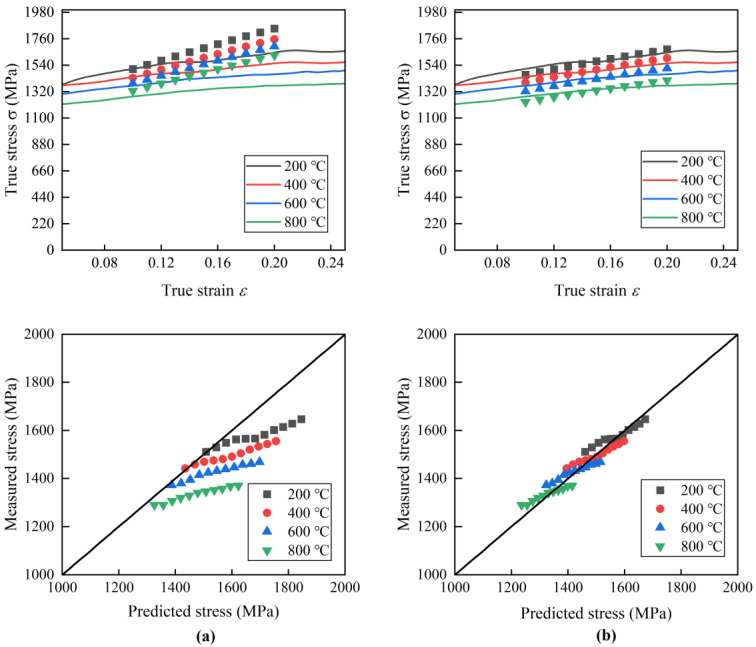
Comparison of modification and measured flow stresses using basic J-C constitutive model and J-C constitutive modified model. (**a**) Basic J-C constitutive model, (**b**) J-C constitutive modified model.

To verify the accuracy of the Ti2AlNb J-C constitutive modified model, four sets of experimental data with a strain rate of 4000 s^−1^ and a temperature range of 200–800 °C were taken from the high-temperature dynamic impact experiment to compare and verify the predicted flow stress with the J-C constitutive model and the J-C constitutive correction model, as shown in Figure 13.

It can be seen that as the strain increases, the flow stress value of the J-C constitutive model gradually exceeds the measured value. This phenomenon means that the material strain hardening rate reflected by the J-C constitutive model is higher than the measured strain hardening rate. As the temperature increases, the difference between the strain rate of the J-C constitutive model and the measured value further increases. The J-C constitutive modified model has a high degree of conformity between the flow stress values and the measured values in the strain range of 0.10–0.20 and the temperature range of 200–800 °C, and the strain hardening rate is consistent with the measured strain hardening rate. According to Equation (17), the maximum relative errors between the predicted and measured values of the basic J-C constitutive model and the modified J-C constitutive model are 14.4% and 3.1%, respectively. It can be concluded that the accuracy of the J-C constitutive modified model based on the strain–strain rate coupling effect is higher than that of the basic J-C constitutive model.

## 5. Conclusions

1. Ti_2_AlNb showed work hardening under quasi-static compression without a significant yield phase; under high strain rate compression, the material’s deformation increased with strain rate, but no fracture occurred. Ti_2_AlNb exhibited the work hardening effect but does not show an obvious yield stage during quasi-static compression test. In the SHPB test, the deformation of specimens increased with the increase in strain rate, and no evident fracture was found.
2. In the SHPB test, Ti_2_AlNb exhibited an evident temperature softening effect and low strain rate sensitivity. The strain rate strengthening effect was detected when the strain rate was 4000–6000 s^−1^. When the strain rate was more than 8000 s^−1^, the strain rate softening effect was detected, and the temperature softening effect also increased with the increase in strain rate.
3. The basic J-C constitutive model could be modified well with the function describing the effect of strain rate on the strain rate strengthening parameters under various temperatures. The relative errors between the experimental measured values and predicted values in various experimental conditions with the modified J-C model were less than 5.0%. The results verified that the modified J-C model could accurately describe the dynamic mechanical properties of Ti2AlNb at high temperatures and strain rates.

Due to the limitation of the SHPB device, the strain rate of the Ti2AlNb dynamic impact experiment reached 12,000 s^−1^, which still lags behind the actual cutting process. The next step is to optimize the SHPB apparatus and conduct numerical simulation and experimental verification to better guide the cutting process.

## Figures and Tables

**Table 1:** Chemical composition of hot forged Ti_2_AlNb alloy (mass).

Element	Al	Nb	Mo	Ti	Ni	Fe	Si	C	O	N	H
fraction%	9.9~11.9	41.6~44.6	≤1.5	Matrix	≤0.10	≤0.30	≤0.10	≤0.10	≤0.10	≤0.02	≤0.01

**Table 2:** Average value of plastic strain of hot forged Ti2AlNb alloy at varied strain rates.

Strain Rate s^−1^	Plastic Strain Range	Medium Strain
4000	0.0579–0.2544	0.1562
6000	0.0537–0.2842	0.1689
8000	0.0250–0.1891	0.1071
10,000	0.0601–0.2006	0.1304
12,000	0.0819–0.2563	0.1691

**Table 3:** Fitted temperature softening index *m* at various strain rates.

**Strain rate (s^−1^)**	4000	6000	8000	10,000	12,000
***m***	1.16	0.73	0.83	0.92	0.80

**Table 4:** Calculated strain rate sensitivity coefficient *C* at specific temperatures and strain rates.

Strain Rate (s^−1^)	Temperature (°C)			
	200	400	600	800
4000	0.00983	0.01638	0.02583	0.03941
6000	0.01998	0.03728	0.056	0.08231
8000	0.01261	0.02647	0.04423	0.06953
10,000	0.01934	0.03215	0.04829	0.07314
12,000	0.03015	0.04592	0.06148	0.07753

**Table 5:** Plastic strain hardening rate at specific strain rates in quasi-static compression and SHPB tests.

**Strain rate s^−1^**	0.001	4000	6000	8000	10,000	12,000
**Strain hardening rate**	2777.75	1581	1502	1483	1409	1354

**Table 6:** Relative errors between the experimental measured stress values and the calculated values at specific strain rate and temperature.

Strain Rate (s^−1^)	Temperature (°C)			
	200	400	600	800
4000	2.2%	3.1%	2.6%	2.5%
6000	2.5%	2.7%	3.3%	3.4%
8000	3.0%	3.5%	3.2%	3.3%
10,000	3.7%	3.6%	3.6%	3.5%
12,000	4.3%	4.6%	5.0%	4.7%

## Data Availability

Data are contained within the article.

## References

[1] Zhang H., Yan N., Liang H., Liu Y. (2021). Phase transformation and microstructure control of Ti2AlNb-based alloys: A review. J. Mater. Sci. Technol..

[2] Goyal K., Sardana N. (2021). Phase stability and microstructural evolution of Ti2AlNb alloys-a review. Mater. Today Proc..

[3] Zhao B., Huang P., Zhang L., Li S., Zhang Z., Yu Q. (2020). Temperature effect on stacking fault energy and deformation mechanisms in titanium and titanium-aluminium alloy. Sci. Rep..

[4] Germann L., Banerjee D., Guédou J.Y., Strudel J.L. (2005). Effect of composition on the mechanical properties of newly developed Ti2AlNb-based titanium aluminide. Intermetallics.

[5] Dressler U., Biallas G., Mercado U.A. (2009). Friction stir welding of titanium alloy TiAl6V4 to aluminium alloy AA2024-T3. Mater. Sci. Eng. A.

[6] Fu Z., Gao G., Wang Y., Qiao H., Xiang D., Zhao B. (2022). Research on dynamic mechanical properties and plastic constitutive relation of Ti3Al intermetallic compounds under mechanical-thermal coupling. J. Mater. Res. Technol..

[7] Cao X., An D., Liu Q., Chen G., Li X. (2024). Precipitation hardening characterization and stress prediction model in electrically-assisted Ti2AlNb uniaxial tension. Intermetallics.

[8] Zhang S., Zhang H., Liu X., Wang S., Wang C., Zhou G., Zhang S., Chen L. (2024). Thermal deformation behavior investigation of Ti–10V–5Al-2.5 fe-0.1 B titanium alloy based on phenomenological constitutive models and a machine learning method. J. Mater. Res. Technol..

[9] Cao Y., Lin X., Kang N., Ma L., Wei L., Zheng M., Yu J., Peng D., Huang W. (2021). A novel high-efficient finite element analysis method of powder bed fusion additive manufacturing. Addit. Manuf..

[10] Ford E., Maneparambil K., Rajan S., Neithalath N. (2021). Machine learning-based accelerated property prediction of two-phase materials using microstructural descriptors and finite element analysis. Comput. Mater. Sci..

[11] Zhao Z.L., Ji H.C., Zhao J.M., Liu B.X., Pei W.C. (2022). Johnson-Cook model for TC4 titanium alloy based on compression experiment. Metalurgija.

[12] Lin H., Jin G., Zhan Q., Wang G., Han J. (2023). Mechanical Properties and Constitutive Model of TC4 Titanium Alloy at Cryogenic. J. Mater. Eng. Perform..

[13] Zhang H., Hu D., Ye X., Chen X., He Y. (2022). A simplified Johnson-Cook model of TC4T for aeroengine foreign object damage prediction. Eng. Fract. Mech..

[14] Ling C., Ren X., Wang X., Li Y., Liu Z., Wang B., Zhao J. (2024). Dynamic Mechanical Properties and Modified Johnson-Cook Model Considering Recrystallization Softening for Nickel-Based Powder Metallurgy Superalloys. Materials.

[15] Hou X., Liu Z., Wang B., Lv W., Liang X., Hua Y. (2018). Stress-strain curves and modified material constitutive model for Ti-6Al-4V over the wide ranges of strain rate and temperature. Materials.

[16] Tian X., Yan K., Zhao J., Wang Q., Wang Y., Chen X. (2022). Properties at Elevated Temperature and High Strain Rate and Establishment of Johnson-Cook Constitutive Model for GH2132. China Mech. Eng..

[17] Lin M., Pang B., Zhang W., Chi R. (2009). Experimental investigation on a dynamic constitutive relationship of 5A06 Al alloy. Explos. Shock. Waves.

[18] Zhou X., Zhao C.M., Li L., Huang H.J. (2014). Numerical simulation of dynamic behavior of extruded AZ91D magnesium alloy based on SHPB experiment. Chin. J. Nonferrous Met..

[19] Hu W.J., Xie R.Z., Huang X.C., Yan Y.X. (2011). Constitutive equation for V-5Cr-5Ti at high temperatures measured using the SHPB technique. Adv. Manuf. Technol..

[20] He Z., Wang Z., Lin P. (2019). A comparative study on Arrhenius and Johnson–Cook constitutive models for high-temperature deformation of Ti2AlNb-based alloys. Metals.

[21] Xue K., Guo S., Ji X., Meng M., Li P. (2023). Investigation on Ultrasonic Vibration Effects on the Plastic Flow Behavior of Ti2AlNb Alloy: Johnson–Cook Model. J. Mater. Eng. Perform..

[22] Sim K.H., Li Y.C., Li C.H., Kim M.O., Kim H.C. (2021). Constitutive Modeling of a Fine-Grained Ti2AlNb-Based Alloy Fabricated by Mechanical Alloying and Subsequent Spark Plasma Sintering. Adv. Eng. Mater..

[23] He L., Su H., Xu J., Liang Z. (2018). Inverse identification of constitutive parameters of Ti2AlNb intermetallic alloys based on cooperative particle swarm optimization. Chin. J. Aeronaut..

[24] He L., Su H., Xu J., Zhang L. (2017). Study on dynamic chip formation mechanisms of Ti2AlNb intermetallic alloy. Int. J. Adv. Manuf. Technol..

[25] Wang Y., Zhou D., Zhou Y., Sha A., Cheng H., Yan Y. (2021). A Constitutive Relation Based on the Johnson–Cook Model for Ti-22Al-23Nb-2 (Mo, Zr) Alloy at Elevated Temperature. Crystals.

[26] (2017). Metallic materials—Compression Test Method at Room Temperature.

[27] (2017). Metallic materials—High Strain Rate Compression Test Method at Ambient Temperature.

[28] Badrish C.A., Morchhale A., Kotkunde N., Singh S.K. (2021). Prediction of flow stress using integrated J-C-ZA constitutive model for Inconel 625 alloy. Mater. Today Proc..

[29] Guo H., Zhou C., Wang K., Jiang Z. (2019). Simulation of ultrasonic vibration cutting performance of GH2132 superalloy. IOP Conf. Ser. Mater. Sci. Eng..

[30] Zhang F., Liu Z., Wang Y., Mao P., Kuang X., Zhang Z., Ju Y., Xu X. (2020). The modified temperature term on Johnson-Cook constitutive model of AZ31 magnesium alloy with {0002} texture. J. Magnes. Alloys.

